# Fourier Transform Infrared (FT-IR) Spectroscopy and Simple Algorithm Analysis for Rapid and Non-Destructive Assessment of Cotton Fiber Maturity and Crystallinity for Plant Mapping

**DOI:** 10.3390/s24092888

**Published:** 2024-04-30

**Authors:** Hee-Jin Kim, Yongliang Liu, Linghe Zeng

**Affiliations:** 1 Cotton Fiber Bioscience & Utilization Research Unit, Southern Regional Research Center (SRRC), Agricultural Research Service, United States Department of Agriculture, New Orleans, LA 70124, USA; heejin.kim@usda.gov; 2 Cotton Quality & Innovation Research Unit, Southern Regional Research Center (SRRC), Agricultural Research Service, United States Department of Agriculture, New Orleans, LA 70124, USA; 3 Crops Genetics Research Unit, Agricultural Research Service, United States Department of Agriculture, Stoneville, MS 38766, USA; linghe.zeng@usda.gov

**Keywords:** fourier transform infrared spectroscopy, attenuated total reflection, algorithm analysis, cotton fiber, node-by-node mapping, maturity, crystallinity

## Abstract

Information on boll distribution within a cotton plant is critical to evaluate the adaptation and response of cotton plants to environmental and biotic stress in cotton production. Cotton researchers have applied available conventional fiber measurements, such as the high volume instrument (HVI) and advanced fiber information system (AFIS), to map the location and the timing of boll development and distribution within plants and further to determine within-plant variability of cotton fiber properties. Both HVI and AFIS require numerous cotton bolls combined for the measurement. As an alternative approach, attenuated total reflection Fourier transform infrared (ATR FT-IR) spectroscopy was proposed to measure fiber maturity (*M*_IR_) and crystallinity (*CI*_IR_) of a sample as little as 0.5 mg lint. Extending fiber maturity and crystallinity measurement into a single boll for node-by-node mapping, FT-IR method might be advantageous due to less sampling amount compared with HVI and AFIS methods. Results showed that FT-IR technique enabled the evaluation of fiber *M*_IR_ and *CI*_IR_ at a boll level, which resulted in average *M*_IR_ and *CI*_IR_ values highly correlated with HVI micronaire (MIC) and AFIS maturity ratio (M). Hence, FT-IR technique possesses a good potential for a rapid and non-destructive node-by-node mapping of cotton boll maturity and crystallinity distribution.

## 1. Introduction

Cotton is one of the most important crops that produces primarily natural fibers for textile and clothing purposes, and also provides edible vegetable oil and cellulosic biomass byproducts as well as protein and fiber sources in animal feedstuff [1]. Cotton boll is the reproductive organ producing cotton fibers and plays an important role in determining cotton fiber yield and quality. Secondary cell walls (SCWs) of cotton fibers are almost exclusively composed of cellulose [2]. As cellulose content increases during cotton fiber developmental stages, the degree of fiber wall thickness (maturity) and crystallinity also increase [3]. Differences in cotton yield and fiber maturity within plant architecture or canopy have been well documented from Upland cotton (*Gossypium hirsutum* L.) plants [4,5,6,7,8]. Upland cotton demonstrates a unique growing pattern of nodes and branches within a cotton plant, allowing determination of the age of each cotton boll based on its location within a plant [9].

Information on boll distribution within a cotton plant is essential for evaluating the adaptation and response of cotton plants to environmental and biotic stress, and for optimizing resource allocation and management strategies in the cotton industry [6]. Cotton scientists map the location and the timing of boll development within a plant, and then determine within-plant variability of cotton fiber maturity by measuring conventional fiber properties such as micronaire (MIC) and maturity ratio (M) [4,7]. MIC value is used for estimating fiber maturity and fineness, and is indirectly determined by measuring air-flow resistance through a plug of cotton fibers of a given weight. The maturity ratio (M_AFIS_) representing the relative amount of cellulose in the fiber cross section is generally determined by an advanced fiber information system (AFIS) measuring the shape of the fibers from two different angles. Both MIC and M_AFIS_ measurement methods require mixed fiber samples of at least 25 bolls picked from all regions of the plant [7]. Thus, traditional mapping of the spatial boll distribution within a plant or across a field is a labor-intensive and time-consuming process for collecting sufficient samples at each node and branch position of cotton plants [6].

Complementary to conventional fiber property measurement methods, we reported an alternative method using attenuated total reflection Fourier transform infrared (ATR FT-IR) spectroscopy and simple algorithm analysis for rapid and non-destructive assessment of cotton fiber maturity and crystallinity [3]. In the analysis, three simple algorithms were applied to estimate IR maturity (*M*_IR_), IR crystallinity (*CI*_IR_), or fiber development (R), based on the intensity ratios of three FT-IR bands at 956, 1032, and 1500 cm^−1^, at 708, 730, and 800 cm^−1^, or at 1236, 1315, and 1800 cm^−1^ [3,10], respectively. Unlike the HVI and AFIS measurements, the ATR FT-IR method needs only a small bundle of cotton fibers as little as 0.5 mg for a routine, rapid, and non-destructive analysis. Although the ATR FT-IR method along with a simple algorithmic strategy has been used in different cotton fiber samples (for example, fibers grown in planta vs. in culture; seed cotton fiber vs. ginned fiber), it has not yet been applied to investigate fiber maturity and crystallinity variations of cotton fibers produced in a single plant. The originality of this study lies in exploring spatial fiber maturity and crystallinity variability or distribution within a cotton plant.

In this study, we would test if the FT-IR and simple algorithm analyses could be implemented for node-by-node mapping and further for fiber maturity and crystallinity evaluation of cotton fibers produced from a single boll located at each node and branch location. Unlike the conventional methods that analyze mixed fiber samples of numerous cotton bolls harvested from multiple plants, the FT-IR method enables the determination of the within-plant fiber variability from each cotton boll harvested from a single plant. Thus, we determined the fiber maturity and crystallinity of each cotton boll harvested from all node and branch positions of three cotton plants for statistical analyses, and also compared them with the corresponding fiber properties measured using the conventional methods. Our results showed that the FT-IR and simple algorithm analyses enabled the evaluation of fiber maturity and crystallinity of cotton fibers harvested from a single boll located at each node and branch position. Further, average *M*_IR_ and *CI*_IR_ values of distributed bolls from three cotton plants were highly correlated with the corresponding micronaire (MIC) and maturity ratio (M) measured via the traditional mapping with conventional fiber measurement methods. Thus, we concluded that ATR FT-IR spectroscopy, combined with a simple algorithm analysis, efficiently monitored fiber maturity and crystallinity of cotton fibers harvested from a single boll and was suitable for node-by-node mapping of cotton boll distribution.

## 2. Materials and Methods

### 2.1. Plant Materials and Growth Conditions

**Figure 1:**
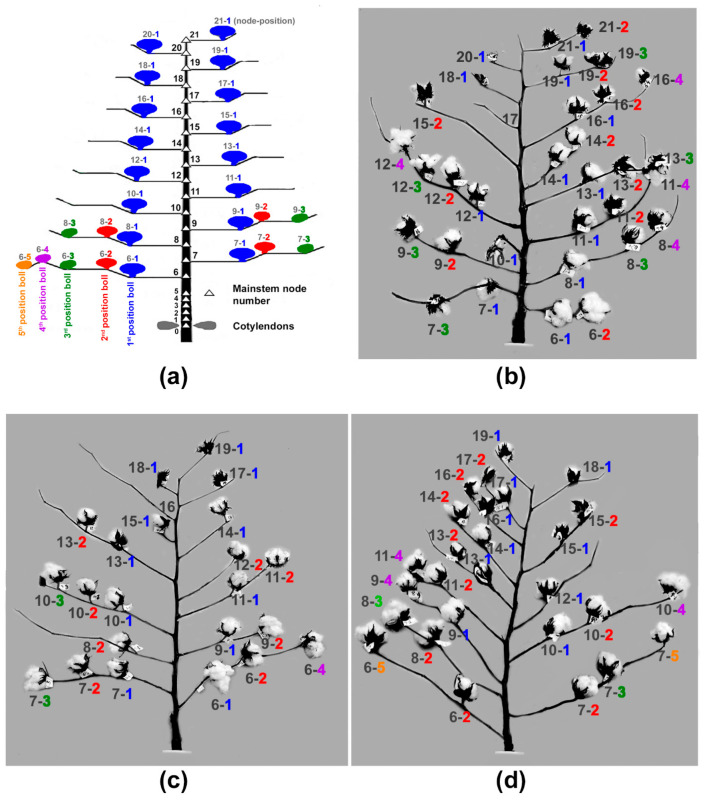
Cotton boll locations based on mainstem nodes and branch positions. (**a**) Illustration of the basic structure of Upland cotton plant. Each cotton boll is generally classified according to mainstem node number and branch position (node number-branch position). Mainstem node number is counted from the cotyledon node counted as node 0. (**b**) Cotton boll locations within the A plant. (**c**) Cotton boll locations within the B plant. (**d**) Cotton boll locations within the C plant. Node numbers were labeled with black font, whereas fruiting position numbers at 1st, 2nd, 3rd, 4th, and 5th position of each branch were labeled with blue-, red-, green-, purple-, and orange-colored fonts, respectively.

A total of 368 plants including the Texas Marker-1 (TM-1, a genetic standard Upland cotton variety) [11], immature fiber (*im*) mutant, and their F_2_ progeny plants were planted on 19 April 2011 at Stoneville (NC, USA) for characterizing fiber maturity. Plants were planted in single-row plots (9.1 m × 1.0 m) with about 45 cm space between plants. Standard conventional field practice and irrigation were applied during the planting season. The soil type was Bosket fine sandy loam. At the end of the growing season, a defoliator (Ginstar, Thidazuron + Diuron) was sprayed on the plants 139 days after planting (DAP) (3 oz) and 146 DAP (6 oz) for removing leaves from the cotton plants. Boll Buster (ethephon) was also applied on 146 DAP to open immature cotton bolls. At the harvest day (156 DAP), three representative TM-1 plants were selected for their typical architectures with maximum intact fruiting branches and nodes, and these plants were named A, B, and C plants. Each boll at every node and branch position on the plants was tagged and photographed (Figure 1b–d).

### 2.2. Agronomic Fiber Property Measurements with Individual Bolls

Each cotton boll was manually collected and individually stored in brown bags. They were kept in a dark storage room with a constant temperature (23 ± 1 °C) and relative humidity (50 ± 10%). The number of open bolls was counted from each plant. As shown in Figure S1, each boll was classified into four components including lint, seeds, bur, and bracts during manual harvesting and laboratory roller gin processing. The mass of seeds and lint were weighed using a balance. The lint percentage (%) of individual bolls was determined by dividing the lint mass by the seed cotton mass, followed by multiplying 100.

### 2.3. ATR FT-IR Spectral Collection and Data Analysis with the Lint Harvested from Individual Bolls

The lint collected from individual bolls was scanned using an FTS 3000MX FT-IR spectrometer (Varian Instruments, Randolph, MA, USA) equipped with a ceramic source, KBr beam splitter, and deuterated triglycine sulfate (DTGS) detector and attenuated total reflection (ATR) attachment. The ATR sampling device utilized a DuraSamplIR single-pass diamond-coated internal reflection accessory (Smiths Detection, Danbury, CT, USA), and a consistent contact pressure was applied by way of a stainless steel rod and an electronic load display. Preparing cotton fiber samples for a regular FT-IR transmission measurement is a rather complex task, mostly because of the time-consuming procedure of grinding the cotton fibers to make the well-known “KBr-pellet”. To overcome the disadvantages of the KBr-pellet method, the ATR device as a rapid, non-destructive, and routine technique has been adopted extensively in cotton fiber FT-IR studies [3,10,12,13,14,15,16]. The use of the ATR device makes fiber sampling easier and simpler than the KBr-pellet method, hence it is time-efficient in analyzing a large number of samples. The sampling depth of the ATR device is from 2 to 15 μm depending on ATR crystal materials and also increases with decreasing wavenumber [17,18]. Since the thickness of the secondary cell wall (SCW) in mature cotton fibers varies from 2 to 7 μm [19], the ATR method is capable of representing the information inside mature cotton fibers with the use of both a low refractive index crystal (i.e., diamond or ZnSe) and a low spectral region (1100–600 cm^−1^).

During the data collection, cautions were taken to make sure that the window (2 mm in diameter) of the ATR sampling device was covered completely by fiber samples. At least five measurements for each sample that was free of any impurities (or non-lint materials), by re-sampling at different locations across the entire sample, were collected over the range of 4000–600 cm^−1^ at 4 cm^−1^ and 16 co-added scans. All spectra were given in absorbance units and no ATR baseline correction was applied. The spectral set was loaded into Microsoft Excel 2016 to execute simple algorithm analyses for assessing fiber *CI*_IR_ and *M*_IR_ indices with the identical procedure reported [3,10], in which a multi-point intensity average at respective wavenumbers (or bands) in absorbance spectra was calculated to represent the specific band intensity.

*M*_IR_ estimation included two equations, with the first algorithmic *R*_1_ equation (Equation (1)) to calculate the *R*_1_ value and the second algorithm *M*_IR_ equation (Equation (2)) to convert the *R*_1_ value into the *M*_IR_ index,

*R*_1_ = (*I*_956_ − *I*_1500_)/(*I*_1032_ − *I*_1500_) (1)

*M*_IR_ = (*R*_1_ − 0.14)/0.45 (2)

where *I*_1500_, *I*_1032_, and *I*_956_ are each a three-point intensity average at respective wavenumbers, and *R*_1_ is the *R*_1_ value for the unknown sample [10].

Similarly, *CI*_IR_ was computed using two separate algorithms, with the first algorithm *R*_2_ utilizing three respective IR intensities at 800, 730, and 708 cm^−1^ (Equation (3)), and the second algorithm *CI*_IR_ (%) changing the *R*_2_ values into fiber *CI*_IR_ (Equation (4)) [10].

*R*_2_ = (*I*_708_ − *I*_800_)/(*I*_730_ − *I*_800_) (3)

*CI*_IR_ (%) = ((*R*_2_ − 1.4)/2.0) × 100 (4)

To calculate both *M*_IR_ and *CI*_IR_ simultaneously from the Excel program, the first step was to export the spectral data format (for example, .spc) into the Excel readable format (for example, .prn) with the use of Grams/AI (Version 9.1, Thermo Fisher Scientific, Waltham, MA, USA), the second step was to import the .prn file into the Excel program and to assign spectral wavenumbers and absorbances into different columns, and the third step was to input the average functions for calculating six intensity averages at 1500, 1032, 956, 800, 730, and 708^−1^ and also to enter the *M*_IR_ and *CI*_IR_ algorithms for computing *M*_IR_ and *CI*_IR_ values.

### 2.4. Conventional Fiber Property Measurements

After the ATR FT-IR measurement, cotton fibers from the same node and positions of the three plants (A, B, and C) were combined and manually blended. Following a 48 h conditioning of these samples at a controlled and standard laboratory (65 ± 2% relative humidity and 21 ± 1 °C temperature), average micronaire (MIC) from three replicates were determined using a Fibronaire instrument (Motion Control Inc., Dallas, TX, USA) and average maturity ratio (M_AFIS_) values with five replications were measured on individualized fibers using USTER^®^ AFIS PRO2 (USTER Technologies Inc., Knoxville, TN, USA) at the Southern Regional Research Center (SRRC, ARS, USDA).

### 2.5. Calculation of Growing Degree Days (GDDs)

Daily temperature records of Stoneville, MS in 2013 were obtained from the Greenville International Airport through a weather history website (https://weatherspark.com (accessed on 19 December 2023)). Growing degree days (GDDs) or heat unit was calculated from the equation [6,20],

GDD = Σ [(Tmax + Tmin/2) − Tt](5)

where Tmax and Tmin are the daily maximum and minimum temperatures, respectively, and Tt is the threshold temperature (60 °F or 15.6 °C) required for cotton growth and development [20].

### 2.6. Statistical Analyses

Statistical analyses including correlation, linear regression, and nonlinear regression were performed with Prism version 10 software (Graph-Pad Software, Inc., San Diego, CA, USA). The correlation coefficient (*r*) was determined by Pearson’s method [21]. Statistical significance was shown at the probability (*p*) levels value under 0.05 *, 0.01 **, 0.001 ***, and 0.0001 ****.

## 3. Results and Discussion

### 3.1. Cotton Boll Distribution and Within-Plant Variability of Cotton Yield and Fiber Quality

As shown in Figure 1a, each cotton boll within a plant can be classified according to the node and branch position as described by Oosterhuis and Jernstedt [9]. The node numbers were labeled with black font, and fruiting position numbers at the 1st, 2nd, 3rd, 4th, and 5th position of each branch were labeled with blue-, red-, green-, purple-, and orange-colored fonts, respectively (Figure 1a–d). The A plant consisted of 16 sympodial (fruiting) branches between 6 and 21 nodes (Figure 1a), while the B plant and the C plant were composed of 14 sympodial branches between 6 and 19 nodes (Figure 1b,c). Commonly, mature cotton bolls were located at the 1st and 2nd positions of most nodes, but they were observed at the 3rd, 4th, and 5th positions of a few nodes.

There were substantial variations in total lint weight (g) per plant and lint percentage (Lint%) among the three plants (Table 1). The A plant generated the most boll number (33 bolls) with the greatest lint mass (78.06 g) and the lowest lint percentage (34.69%) among the three plants. The B plant produced the least boll number (22 bolls) with the least lint mass (47.99 g) but the highest lint percentage (36.97%). The C plant produced intermediate boll number (27 bolls), lint mass (70.73 g), and percentage (36.22%) between the A and B plants.

From the aspect of single boll level, the lint mass showed a broad range within the A plant (0.93–3.23 g), B plant (1.36–3.19 g), and C plant (0.84–3.55 g). Mean lint mass and lint percentage of the A plant (2.36 ± 0.67 g and 34.45 ± 1.49%), B plant (2.18 ± 0.43 g and 36.95 ± 1.37%) and C plant (2.62 ± 0.67 g and 35.97 ± 2.03%) also showed a significant (*p* < 0.0001 ****) variation. Abscission due to physiological and environmental factors was suggested to be the main cause generating the variations of boll distribution and lint weight per plant among cotton plants [22,23].

### 3.2. Environmental and Managemental Effects on Cotton Fiber Maturation of Each Cotton Boll

**Figure 2:**
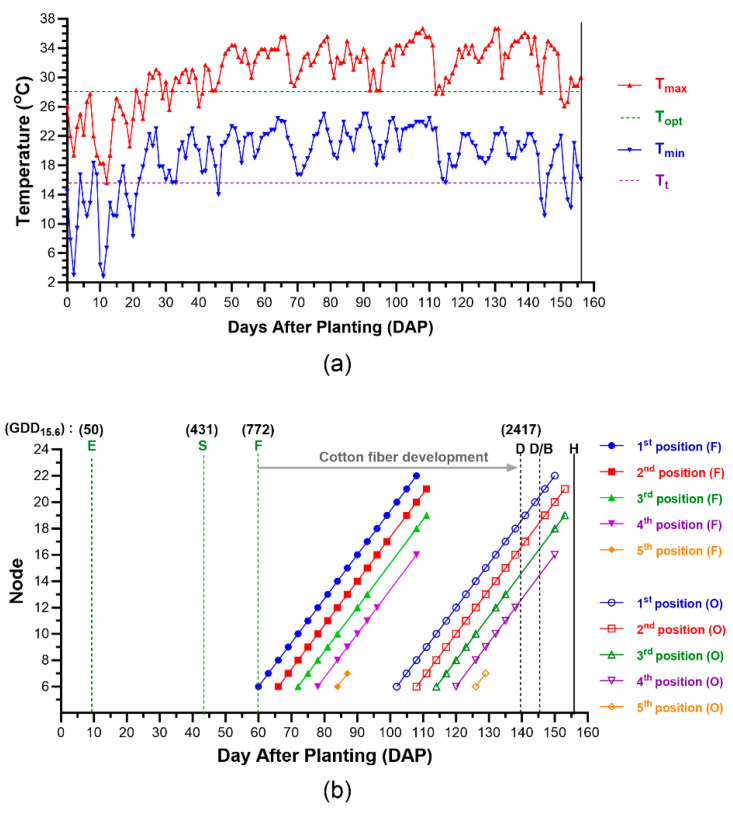
Determination of fiber development stage of the distributed cotton bolls within plants. (**a**) Daily temperature record of the cotton growing season. Daily maximum and minimum temperatures (Tmax and Tmin) as well as the optimum and threshold temperatures (Topt and Tt) for cotton plant growth were described. (**b**) GDD determination. Growing degree days (GDDs) were calculated from the individual bolls located at the nodes (6th to 21st) and fruiting position (1st to 5th) of the three plants. Planting, sprays for a defoliator (D) and a boll opener (B), and harvesting (H) were performed on 0, 139, 146, and 156 DAP, respectively. Occurrences of emergency (E), squaring (S), and flowering (F) were determined based on the GDD and DAP based on the daily temperatures and the method described by Oosterhuis [22].

On the irrigated cotton field, conventional practices were performed for growing the cotton plants. Planting, sprays of a defoliator (D) and a boll opener (B), and harvesting (H) were performed on 0, 139, 146, and 156 DAP, respectively (Figure 2). As shown by Oosterhuis [22], the flowering interval between the two adjacent positions at the same node was 6 days and the flowering age difference between the two adjacent nodes at the same fruiting position was 3 days. Growing degree days (GDDs) of the cotton growing season were calculated using the daily temperatures in the cotton field (Figure 2a). According to the designated GDD values for the occurrences of emergency (E), squaring (S), flowering (F), and harvesting (H) [9,22], cotton seedlings emerged on 9 DAP (GDD, 50), and the first flower squares were presented on 43 DAP (GDD, 431). The first flowers were observed on node 6 at the first branch position on 60 DAP. Cotton boll development at most nodes and branch positions took 6 weeks.

Figure 2b also showed that the cotton fiber maturation process of the cotton bolls located at 20-1 could be mildly affected by the first defoliator (3 oz) spray on 139 DAP. Furthermore, cotton fiber maturation at several cotton bolls at the 1st position (20-1 and 21-1), the 2nd position (19-2, 20-2, and 21-2), the 3rd position (18-3 and 19-3), and the 4th position (16-4) could be severely affected by the second and major defoliator (6 oz) as well as boll buster that was treated on 146 DAP.

These results demonstrated that the GDD value was a good indicator representing the climate conditions experienced by the cotton plants. In addition to the environmental factors, a management factor like the defoliation timing also affected cotton fiber growth and development which ultimately affected cotton yield and fiber quality.

### 3.3. Determination of Fiber Maturity and Crystallinity from a Single Boll Using FT-IR Spectroscopy

#### 3.3.1. ATR FT-IR Spectral Characteristics of Cotton Fibers Harvested from a Single Boll at Various Nodes and Positions within a Plant

**Figure 3:**
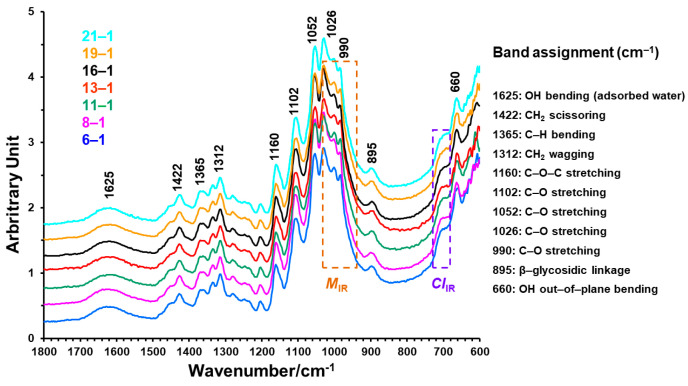
Representative ATR FT-IR spectra of single boll fibers from the 1st position of differing nodes in the A plant. These spectra were normalized by dividing the intensity of the individual band in the 1800–600 cm^−1^ region with the average intensity in this 1800–600 cm^−1^ region. Spectra were shifted vertically for visualization. Key spectral regions for *M*_IR_ and *CI*_IR_ estimation were shown in two rectangular areas.

Figure 3 compares the ATR FT-IR spectral intensity variations in the 1800–600 cm^−1^ region among single cotton boll fibers from the 1st position of different nodes (6-1, 8-1…21-1) within the A plant. There exist similarities in both band shapes and positions visually between these fibers. This observation, reasonably, differs from apparent spectral intensity increases or decreases in this region for developmental TM-1 cotton fibers collected at different DPA [3]. Earlier studies [3,10] have assigned a broad band centered at 1620 cm^−1^ to the O–H bending mode of adsorbed water, bands at 1422, 1366, and 1312 cm^−1^ to C–H vibrations, at least five intense bands in the 1200–1000 cm^−1^ region to the stretching modes of C–O vibrations, a band at 895 cm^−1^ to the β-glycosidic linkage in cellulose, and weak bands from 800 to 700 cm^−1^ to crystal forms of native cellulose in cotton fibers. Obviously, the bands below 1500 cm^−1^ are complicated with many unassignable overtone and combination modes. In addition, there were intense absorptions between the 3600 and 2750 cm^−1^ region that are assignable to the hydrogen-bonded O–H stretching vibrations as well as methylene (CH_2_) and methine (=CH-) functional group C–H stretching vibrations (not shown).

#### 3.3.2. IR Maturity Variation among Individual Bolls within Cotton Plants

**Figure 4:**
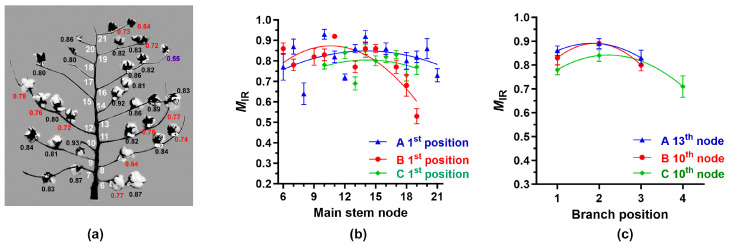
Variation of the *M*_IR_ values measured from a single boll located at different nodes and positions of the three plants. (**a**) *M*_IR_ values of each boll of the A plant. *M*_IR_ values of mature fibers (>0.80), intermediately mature fibers (0.59–0.80), and immature (<0.59) fibers [10] were labeled with black, red, and purple, respectively. (**b**) *M*_IR_ value at the 1st positions with various nodes among the three plants (A, B, and C). (**c**) *M*_IR_ values at various positions among the three plants. The second polynomial regressions were applied to (**b**,**c**). The error bar represents the standard error of the mean (SEM).

*M*_IR_ values were determined from the cotton fibers of each boll by analyzing ATR FT-IR spectra with the simple algorithm proposed before. The *M*_IR_ values of cotton bolls within the A (0.64–0.93), B (0.53–0.92), and C (0.69–0.85) showed a wide range of maturity distribution (Figure 4a), although average *M*_IR_ values of the cotton bolls of the A (0.82), B (0.79), and C (0.79) plant appeared to be similar to one another. The *M*_IR_ values at the 1st position bolls increased from the bottom 6th node to the middle nodes (10-15) of each plant, and the *M*_IR_ values reduced to the terminal nodes (19-21) as shown in Figure 4a. To fit the curves, we tested three models including the second-order polynomial (quadratic), simple linear, and exponential equation. Among them, the second-order polynomial model shown in Figure 4 provided the best fit based on the R^2^ value. It showed significant (*p*, 0.006 **) variations of the *M*_IR_ values located at the 1st positions of each node among the three plants. The *M*_IR_ values increased from the 1st to the 2nd position and reduced to the 4th position (Figure 4b). It showed significant (*p*, 0.0008 ***) variations of the *M*_IR_ values at the various positions among the three plants.

Consistent with the GDD results (Figure 2b), the A plant showed relatively lower *M*_IR_ values at the terminal node at 21-2 (0.64) and also at the terminal position at 16-4 (0.55) and 19-3 (0.72), in which their fiber developments were inhibited by the defoliator treatments (Figure 4a). Similarly, the B plant showed relatively lower *M*_IR_ values at the terminal node 18-1 (0.68) and 19-1 (0.53). Hence, we concluded that the *M*_IR_ values determined by ATR FT-IR and simple algorithm approach showed the variability of fiber maturity within and among cotton plants, and these patterns were similar to the variations of cotton mass shown in Table 1.

#### 3.3.3. IR Crystallinity Variation among Individual Bolls within Cotton Plants

**Figure 5:**
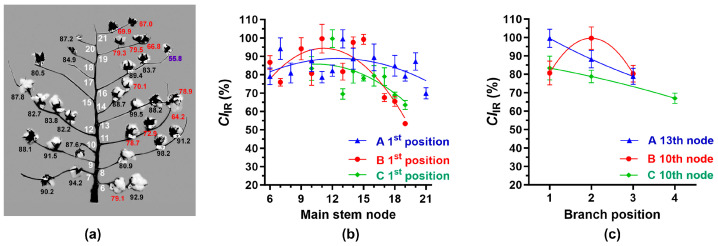
Variation of the *CI*_IR_ values measured from a single boll located at different nodes and positions of the three plants. (**a**) *CI*_IR_ values of each boll of the A plant. High (>80%), middle (59–80%), and low (<59%) *CI*_IR_ values [10] were labeled with black, red, and purple, respectively. (**b**) *CI*_IR_ value at the 1st positions with various nodes among the three plants (A, B, and C). (**c**) *CI*_IR_ values at various positions among the three plants. The second polynomial regressions were applied to (**b**,**c**). The error bar represents the standard error of the mean (SEM).

The *CI*_IR_ indices of cotton bolls within the A (55.8–98.2%), B (53.4–99.6%), and C (53.8–92.2%) plants showed a wide range of variations (Figure 5a). Average *CI*_IR_ values of the cotton bolls at the 1st positions from the 6th to 21st nodes were 84.7% (A plant), 82.0% (B plant), and 78.1% (C plant). The *CI*_IR_ values at the 1st positions increased from the bottom 6th node to the middle nodes (10th–15th) of each plant, and the *CI*_IR_ values reduced to the terminal nodes (19th–21st) as shown in Figure 5a. It showed significant (*p*, 0.0135 *) variations of the *CI*_IR_ values located at the 1st positions among the three plants. Similarly, the *CI*_IR_ values at the terminal 3rd or 4th position were lower than the 1st or 2nd position. It showed significant (*p*, 0.0144 *) variations of the *CI*_IR_ values at the various positions among the three plants (Figure 5b).

Consistent with the GDD results (Figure 2b), the A plant showed relatively lower *CI*_IR_ values at the terminal node at 21-1 (69.9%) and 21-2 (67.0%) as well as the terminal position at 16-4 (55.8%) and 19-3 (66.8%), whose fiber development was inhibited by the defoliator treatments (Figure 5a). Similarly, relatively lower *CI*_IR_ values were detected at the terminal nodes including 18-1 (65.5%) and 19-1 (53.4%) nodes from the B plant, and 17-2 (53.8%), 18-1 (68.9%), and 19-1 (63.5%) from the C plant.

**Figure 6:**
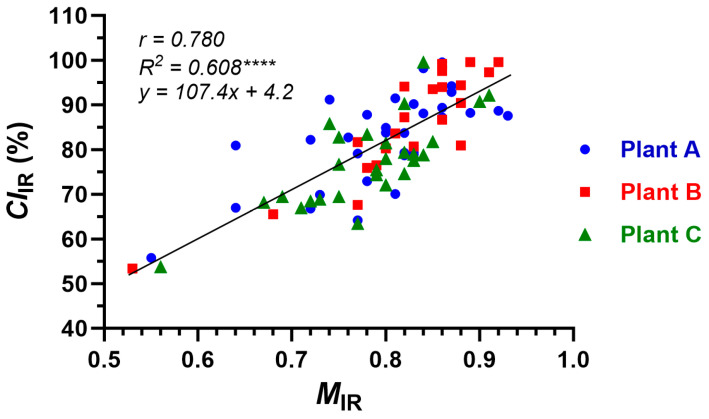
Relationships between IR maturity (*M*_IR_) and IR crystallinity (*CI*_IR_) determined from individual bolls of the three plants (A, B, and C). **** *p*-value < 0.0001 (very significant).

Comparisons of *M*_IR_ and *CI*_IR_ values calculated from Equations (2) and (4) on the three plants (*r*, 0.780; *R*^2^, 0.608) showed significant (*p* < 0.0001 ****) linear regression patterns (Figure 6). The results indicated a within-plant variability of both maturity and crystallinity, and a pattern of more mature fibers tending to have higher crystallinities.

### 3.4. Comparisons of Fiber Properties Measured between FT-IR Spectroscopy and Conventional Methods with the Combined Samples at the Same Positions of the Three Plants

#### 3.4.1. Determination of Fiber Properties via Conventional Methods

Micronaire (MIC) is a conventional fiber property estimating a combination of fiber maturity and fineness, and measured using Fibronaire or high volume instrument (HVI) requiring a minimum of 3.24 or 10 g of cotton fibers, respectively [24]. Because a single boll of any plant produced 2.18–2.62 g fiber (Table 1), direct MIC measurements of fibers at a single boll level for a node-by-node mapping are infeasible. Hence, we combined the cotton fibers from the same nodes and positions among the three plants, and measured the MIC and M values. In practice, collecting numerous cotton bolls at each node and position for the conventional fiber measurement methods are labor-intensive and time-consuming processes.

**Figure 7:**
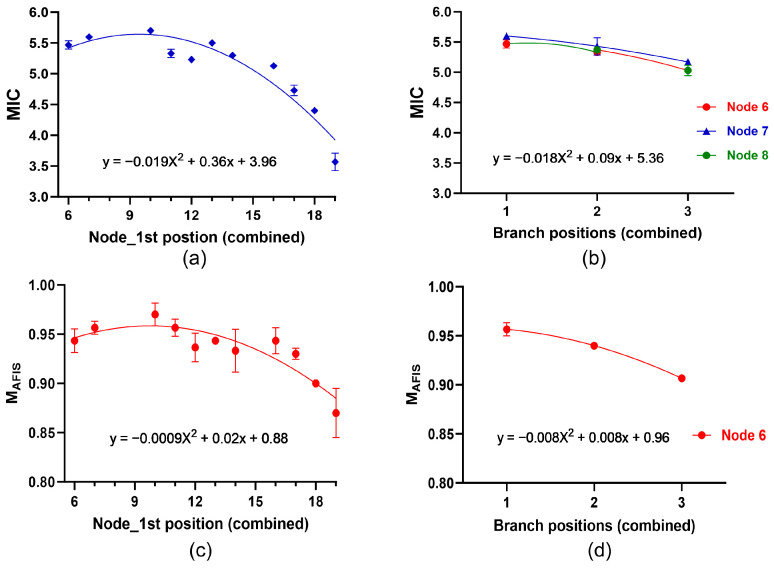
Within-plant variability of cotton fiber properties measured from the combined fibers of the same locations of the three plants (A, B, and C) using conventional methods. (**a**) MIC values of the combined fibers collected from the 1st positions with different nodes. (**b**) MIC values of the combined fibers at the different positions of the 6th node. (**c**) M_AFIS_ values of the combined fibers collected from the 1st positions with different nodes. (**d**) M_AFIS_ values of the combined fibers at the different positions of the 6th node. The second polynomial regressions were applied to (**a**–**d**). The error bar represents the standard error of the mean (SEM).

The MIC values of the combined fibers showed a broad range between 3.57 and 5.70, and the mean MIC value was 4.43. The MIC values at the 1st positions between the 6th and 16th nodes were consistently higher than 5.00, whereas the MIC at the 1st positions between the 16th and 19th nodes were reduced from 5.13 to 3.57 (Figure 7a). The average MIC values also decreased from the 1st (5.54) to the 2nd (5.38), and to the 3rd position (5.10) as shown in Figure 7b.

Maturity ratio (M_AFIS_) is another conventional fiber property estimating fiber maturity that is generally measured with an AFIS instrument in the textile industry [24]. The M_AFIS_ values measured using an AFIS from the combined fibers showed a broad range between 0.870 and 0.957, and the average M_AFIS_ value was 0.935. The M_AFIS_ values at the 1st positions between the 6th and 16th nodes were higher than 0.930, whereas the M_AFIS_ at the 1st positions between the 16th and 19th nodes were reduced from 0.930 to 0.870 as shown in Figure 7c. The M_AFIS_ values also decreased from the 1st (0.957) to the 2nd (0.940), to the 3rd (0.907) position of the same node (Figure 7d).

#### 3.4.2. Determination of Average *M*_IR_ and *CI*_IR_ at Each Node and Position among the Plants

**Figure 8:**
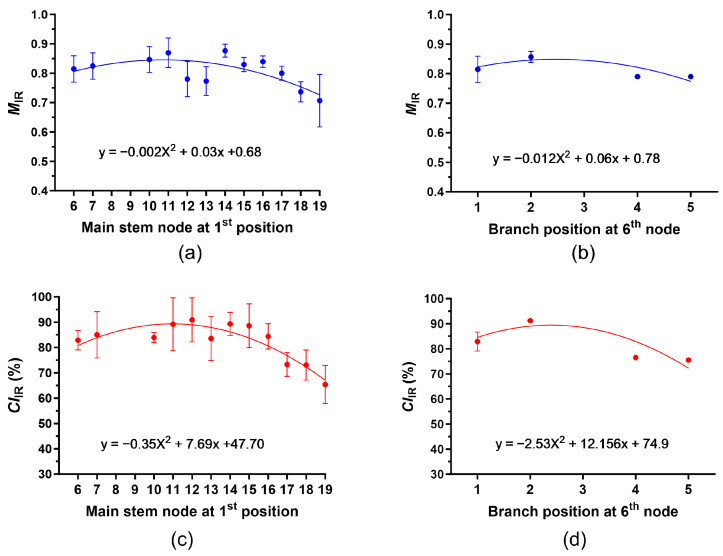
Determination of mean *M*_IR_ and *CI*_IR_ values from cotton fibers collected from the same locations of the three plants (A, B, and C) using ATR FT-IR spectroscopy. (**a**) Mean *M*_IR_ values of the cotton fibers collected from the 1st positions with different nodes. (**b**) Mean *M*_IR_ values of the fibers at the different positions. (**c**) Mean *CI*_IR_ values of the fibers collected from the 1st positions with different nodes. (**d**) Mean *CI*_IR_ values of the fibers at the different positions of the 6th node. The second polynomial regressions were applied to (**a**–**d**). The error bar represents the standard error of the mean (SEM).

Based on the *M*_IR_ and *CI*_IR_ values measured from a single boll located at various nodes and positions of each plant (Figure 4 and Figure 5), we calculated the mean *M*_IR_ and *CI*_IR_ values at each node and position among the three plants (Figure 8).

The maximum mean *M*_IR_ value (0.877) was found at the 1st position of the 14th node, whereas the minimum mean *M*_IR_ value (0.707) was detected at the 1st position of the 19th node. Like the increasing and decreasing pattern of the MIC and M_AFIS_ values, the mean *M*_IR_ values increased from the 6th to 16th nodes, and then decreased to the 19th node (Figure 8a). The mean *M*_IR_ values of the 1st (0.815) and 2nd (0.857) positions were greater than those of the 4th (0.790) and 5th (0.790) positions of the same node (Figure 8b).

The maximum and minimum values of the mean *CI*_IR_ were detected at the 1st position of the 12th (90.9%) and the 19th (65.6%) nodes (Figure 8c). The increasing and decreasing patterns of the mean *CI*_IR_ were almost identical to those of the mean *M*_IR_ values. The mean *CI*_IR_ values between the 6th and 16th nodes were greater than those between the 17th and 19th nodes. Similarly, the mean *CI*_IR_ values of the 1st (82.9%) and 2nd (91.2%) positions were higher than those of the 4th (76.5%) and 5th (75.5%) positions of the same node (Figure 8d).

#### 3.4.3. Relationships between IR Properties and Conventional Maturity Properties

**Figure 9:**
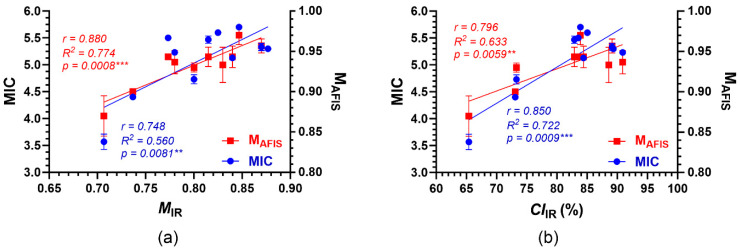
Relationships between IR properties and conventional maturity properties. (**a**) Relationships of mean *M*_IR_ with corresponding MIC and M_AFIS_. (**b**) Relationships of mean *CI*_IR_ with corresponding MIC and M_AFIS_. The error bar represents the standard error of the mean (SEM). ** *p*-value < 0.01 (significant); *** *p*-value < 0.001 (very significant).

The mean *M*_IR_ showed significant and positive correlations with the corresponding MIC (*r*, 0.748; *R*^2^, 0.560 **) and M_AFIS_ (*r*, 0.880; *R*^2^, 0.774 ***) that were measured from the combined fibers via conventional methods (Figure 9a). Similarly, the mean *CI*_IR_ also demonstrated significant and positive correlations with the corresponding MIC (*r*, 0.850; *R*^2^, 0.722 ***) and M_AFIS_ (*r*, 0.796; *R*^2^, 0.633 **) (Figure 9b). When ׀*r*׀ value is less than 0.3, between 0.3 and 0.7, or greater than 0.7, a weak, moderate, or strong (either positive or negative) linear correlation, respectively, exists between the two variables [25]. The results confirmed that either *M*_IR_ or *CI*_IR_ can be used to assess the quality of the cotton fiber samples that were collected for node-to-node mapping assay.

## 4. Conclusions

Direct fiber property measurements for node-by-node mapping are challenging tasks due to the labor-intensive and time-consuming processes of collecting numerous cotton bolls at each node and position for the conventional fiber measurement methods. With the use of an ATR micro-sampling device and simple algorithm analysis, we demonstrated that ATR FT-IR spectroscopy enabled determining fiber maturity and crystallinity at a boll level for node-by-node mapping. Comparing the fiber *M*_IR_ or *CI*_IR_ averages on individual bolls to corresponding HVI MIC and AFIS M_AFIS_ on combined bolls showed a high correlation between the new method and the traditional methods. Results demonstrated the suitability of the ATR FT-IR method in node-by-node mapping of fiber maturity and crystallinity within a plant, and its further application in fiber maturity and crystallinity distribution comparison between cotton plants. Further studies with various environmental conditions (irrigation, rainfalls, and water deficiency) and different levels of major and minor fertilizers would provide information on how cotton fiber development and growth including maturity and crystallinity are affected by those environmental and managemental factors.

## Figures and Tables

**Table 1:** Agronomic lint properties of the three cotton plants.

Classification	Property	A Plant	B Plant	C Plant
Total	Boll number	33	22	27
	Lint mass	78.06 g	47.99 g	70.73 g
	Lint%	34.69%	36.97%	36.22%
Single boll	Mean lint mass (g)	2.36 ± 0.67 g	2.18 ± 0.43 g	2.62 ± 0.67 g
	Lint%	34.45 ± 1.49%	36.95 ± 1.37%	35.97 ± 2.03%

## Data Availability

All data are presented here and are available upon request.

## Supplementary Materials

The following supporting information can be downloaded at https://www.mdpi.com/article/10.3390/s24092888/s1, Figure S1: Components of a cotton boll.
