# Supplementary material for: Fourier Transform Infrared (FT-IR) Spectroscopy and Simple Algorithm Analysis for Rapid and Non-Destructive Assessment of Cotton Fiber Maturity and Crystallinity for Plant Mapping
Source: Sensors (Basel). 2024 Apr 30;24(9):2888. doi: 10.3390/s24092888 (PMC11086078; doi:10.3390/s24092888)
Supplement: Supplementary file 1 [file sensors-24-02888-s001.zip › sensors-2952089-supplementary.pdf]

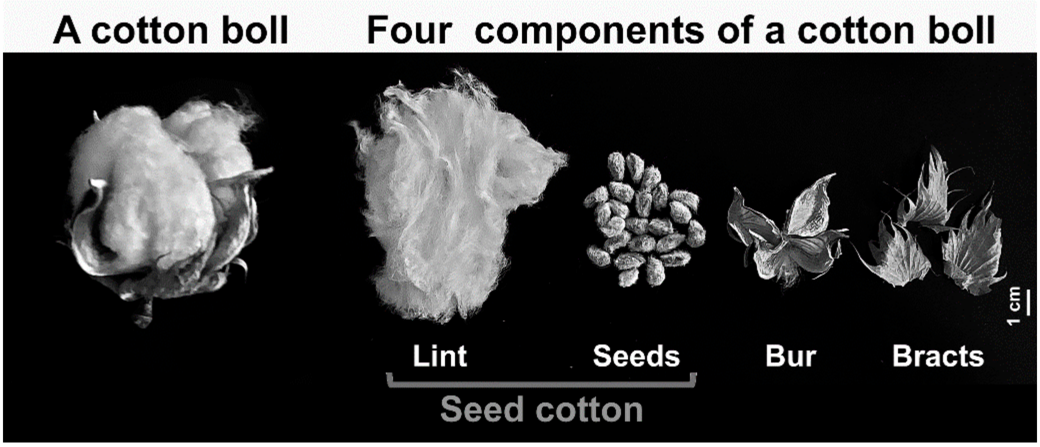


**Figure S1**. Components of a cotton boll. From a single cotton boll (cotton fruit), seed cotton (cotton seeds with associated lint fibers) was harvested manually from a cotton capsule consisting of a bur and bracts. Lint fibers were separated from seeds using a roller gin. “Cotton boll mass” was the combined weight of lint, seeds, bur, and bracts. “Seed cotton mass” was the combined weight of lint and seeds. “Lint mass” was the weight of the ginned fibers. “Lint percentage” (%) of individual bolls was determined by dividing the lint mass with the seed cotton mass, followed by multiplying 100.
